# Reduced Anxiety Associated to Adaptive and Mindful Coping Strategies in General Practitioners Compared With Hospital Nurses in Response to COVID-19 Pandemic Primary Care Reorganization

**DOI:** 10.3389/fpsyg.2022.891470

**Published:** 2022-06-09

**Authors:** Enrico Perilli, Matteo Perazzini, Danilo Bontempo, Fabrizio Ranieri, Dina Di Giacomo, Cristina Crosti, Simona Marcotullio, Stefano Cobianchi

**Affiliations:** ^1^Public Health, Life and Environmental Sciences, Department of Clinical Medicine, University of L’Aquila, L’Aquila, Italy; ^2^Ospedale Regionale San Salvatore dell’Aquila, L’Aquila, Italy; ^3^Atanor Institute of Psychotherapy, L’Aquila, Italy

**Keywords:** anxiety, mindful, coping strategies, general practitioners, hospital nurses, COVID-19

## Abstract

COVID-19 pandemic imposed psychosocial stress increasing in frontline healthcare workers, who managed by responding with different coping strategies. General practitioners were targeted by an extraordinary increase in the demand for reception, diagnosis and treatment from all patients even if working in solo. In Italy, the emergency changed risk assumption and roles in between primary care, unraveling the emotional distress of general practitioners, who suffered not only for isolation, but also emotional threatens. In this correlational study we wanted to evaluate trait anxiety and stress as perceived by general practitioners working in individual ambulatory practice room, and by hospital ward nurses working in group, during a chronic phase (February–May 2021) of COVID-19 pandemic. Our hypothesis is that a different work social organization in clinic contest as for general practitioners compared with nurses could induce adaptive or non-adaptive coping to stress under emergency and mindful attitude could be crucial. A number of 37 general practitioners, and 36 nurses were taken from the sanitary district of ASL1 Avezzano-Sulmona-L’Aquila in Italy. For our analyses we used the Health Professions Stress and Coping Scale to assess the risk of burn-out, and detect the coping strategies. We also used the Cognitive and Affective Mindfulness Scale-Revised, investigating whether clinicians used an eventual mindful attitude to prevent anxiety and responding with adaptive coping strategies. General practitioners reported high levels of anxiety, associated to an increased use of emotional distress. Mindful attitude was protective for anxiety in both general practitioners and nurses. As anxiety increased, it was coped by increasing the demand for social support. This coping strategy correlated with emotional distress and when enhanced, it corresponded to avoidance of the problem. Mindful attitude addressed general practitioners to adaptive coping strategies as the solution of the problem. On the other side, nurses accepted the problem but addressed it to others, by avoiding solving it themselves as beyond their role and organizational responsibility. In conclusion, mindful attitude can prevent dysfunctional reactive behaviors among clinicians at the forefront of emergency and reduce emotional distress for isolation as suffered by general practitioners.

## Introduction

The COVID-19 pandemic has imposed unprecedented challenges on healthcare systems globally. Governments around the world declaring sanitary emergency sudden introduced safety procedures such as quarantine and social distancing, with impacts on the mental health and psychosocial wellbeing of healthcare workers (Babore et al., 2020; Zhang et al., 2020). While it has been demonstrated that healthcare workers have higher resilience scores than general population (West et al., 2020), they face unique workplace demands and are at increased risk of depression, burnout and suicide during daily life outside of crises (Shanafelt et al., 2012; Milner et al., 2016). During the COVID-19 pandemic, frontline healthcare workers have reported even higher levels of anxiety, depression, and post-traumatic stress disorder (Smallwood et al., 2021a). They managed and responded to the psychosocial stress of the pandemic by using different types of coping strategies, which resulted in some cases as adaptive and in others not (Smallwood et al., 2021b). At practice level, during the pandemic all the primary care has altered dramatically, with staff—clinical and administrative—adapting to new socially distanced ways of working for the high risk of infection. Roles and responsibilities of clinicians had to be quickly reorganized on the basis of government decrees for the ongoing health emergency. However, studies have not addressed differences in risk assumption and roles in between primary care, focusing more on frontline nurses and on medical doctors of hospital wards treating COVID-19 symptomatic or severe patients, but less on general practitioners (GPs), who were targeted by an extraordinary and exponential increase in the demand for reception, diagnosis and treatment from all symptomatic and asymptomatic patients, thus exposed to the same frontline risk and demand as ward nurses (Li and Zhu, 2020).

GPs in China as in other countries were at the forefront of tackling the spread of the virus. In general GPs make up 80% of primary care (private or public community) services in most healthcare systems and are prominently at the front-line. They were the first touchpoint of cases presenting with early upper respiratory tract infection symptoms (Low et al., 2021). They received patients providing medical treatment accordingly, as well as conduct surveillance testing of COVID-19. When a COVID-19 positive case was detected, GPs contacted the relevant agencies to transport the patient to an identified isolation facility for further treatment and management. In Italy, GPs is a freelancer affiliated with the national health system. GPs in Italy are also called “family doctors” as they usually accept and follow up patients from the same neighborhood, village or urban area. Most of them are old acquaintances having long taken care of all the members of a family, therefore working and living in great confidence with citizens. Under pandemic the Italian national health system ordered GPs for the therapeutic follow-up of all their patients infected with COVID-19 or symptomatic, whether hospitalized at home, discharged from hospitals or asymptomatic and non-hospitalized. Meanwhile the Italian government and health system informed the citizens not to go directly to the hospitals but first to call the COVID-19 centers or their family doctor for a visit or just for general information. Then GPs suffered not only from an increase in the demand for help, but also for all fears and anxieties of their long time patients. GPs as “family doctors” were the first container of physical and psychological distress of COVID-19 in their often small ambulatory practice room and without having the same spaces, diagnostic tools, assistance and organized group to cope as in hospitals. In comparison to the ward clinicians and nurses at the forefront with pandemic patients, Italian GPs felt left alone in front of patients and reported it several times to journalists and in the Italian Federation of General Practitioners (FIMMG), with several interview articles published on 2020 national newspapers. The mental state of GPs in response to an infectious disease outbreak, especially to one on such a massive scale as the COVID-19 pandemic, is of great concern. There is a consensus in studies examining the wellbeing of doctors during COVID-19 that doctors are at higher risk of suffering from an acute stress reaction, burnout, insomnia, anxiety, depression, and post-traumatic stress disorder, compared to the general population. These have long-term psychological implications (Raudenská et al., 2020; Spoorthy et al., 2020).

Based on Lazarus and Folkman (1984), coping could be defined as the behavioral and cognitive efforts that people use to manage the internal and external demands due to a stressful situation. Although there are many specific coping strategies, researchers have typically conceptualized coping using one of the following two superordinate distinctions: on the one hand (a) problem-focused (addressing external demands of stressors) vs. emotion-focused (addressing internal demands of stressors); on the other hand (b) approach (acting on the demands of a stressor) vs. avoidance (avoiding or disengaging from the demands of a stressor (Suls and Fletcher, 1985).

We have reviewed the stress assessments of clinicians, taking into account also the perspective of the Lazarus and Folkman’s model. Online surveys were conducted to explore the psychological stress status and psychological care needs of the healthcare workers during the COVID-19 outbreak period (Smallwood et al., 2021b); we particularly focused on results from surveys in Europe (Hummel et al., 2021) and Italy (Conti et al., 2020). A considerable proportion of participants showed high values for depression, anxiety, and stress. Even though medical professionals exhibited less mental stress than non-medical professionals, both doctors’ and nurses’ shared common coping strategies to deal with this unusual situation, as “taking protective measures” and “actively acquiring more knowledge about COVID-19,” but these strategies were not sufficient to reduce the observed mental disease. Another important strategy was “video-chatting with family and friends to share concerns and support,” which had high priority for participants. This has the advantage to get in touch directly with people experiencing mental burden, with the help of so-called e-mental health applications. Were observed, the increasing of request for a kind of psycho-social support seemed to be more effective in reducing stress and anxiety. Healthcare workers who perceived the need for psychological support scored above the clinical alarming level in psychological scales. Results from these surveys point out the importance to consider the psychological impact of COVID-19 on Italian health-care workers and strongly suggest providing adequate professional care, training and prevention of post traumatic stress and mental disease, for which there are still no data results for the medium and long term of COVID-19 pandemic.

Few studies have focused on the coping strategies of GPs during the COVID-19 crisis, suggesting that different strategies could be necessary at different phases in the coping of GPs. One study reported that task-oriented management was preferred by GPs (Di Monte et al., 2020), another found a balance of individual and organization strategies, including psychological intervention or support programs that could be useful in mitigating the psychological impact of crisis (Heath et al., 2020). Elsewhere, GPs’ distress and adaptation to crisis was analyzed respect to resilience and emotional response (Seçer et al., 2020; Low et al., 2021), concluding that the positive mentality of the doctors could be considered as a major adaptive coping strategy in the broader context of the prevailing management of the pandemic in the local setting, with a supportive environment and a sense of security and trust as probably the effective management of crisis by the local authorities contributing to positive mental health in the GPs at the frontline. These indications are in line with results of a survey on Italian general population (Tintori et al., 2020), indicating that two coping indicators—problem-oriented and focused on positive emotions coping strategies—were selected as objective variables in a “decision tree” modeling, showing a link between individual factors (i.e., atmosphere at home) and educational and social factors (i.e., compliance with restrictions during the health emergency).

In this correlational study we investigated Italian GPs and hospital COVID-departments nurses anxiety, stress and coping even related to mindfulness and emotion regulation under pandemic, in order to highlight if there are differences induced by role and responsibility in frontline healthcare as first contact point of patients and eventually as following reorganization of primary healthcare under emergency. By using the Cognitive and Affective Mindfulness Scale-Revised (CAMS-R) (Feldman et al., 2006), we investigated whether mindful attitude or training were important strategies as personal resource or psychosocial intervention to prevent anxiety and deal with patients at the first touchpoint under COVID-19 pandemic. Mindfulness indicates an attitude and state of mind connected with attention and awareness useful to cope with stress. Many studies highlighted positive correlations between high mindfulness disposition and psychological well-being, and there is evidence for mindfulness training as an efficient method of distress reduction in medical and psychiatric populations, as well as in non-clinical populations (Baer, 2003; Grossman et al., 2004), were self-report measure of mindfulness is a method to define the coping strategies included in this construct (Dimidjian and Linehan, 2003; Bishop et al., 2004). Mindfulness-based cognitive therapy and stress reduction improve mental health and wellbeing by modulating cognitive and emotional reactivity to stressors, with rumination and worry as significant mediators of mental health outcomes (Gu et al., 2015), and training mindfulness abilities may contribute to making effective coping strategies in clinical and psychological conditions (Chi et al., 2018; Hernando et al., 2019; Perilli et al., 2020). A recent important study investigated the influence of mindfulness training on mental health during the COVID-19 outbreak in China (Zhu et al., 2021), finding lower scores of pandemic-related distress in mindfulness practitioners compared to non-practitioners. Health care professionals report higher levels of mental distress and lower levels of life satisfaction than general population, and they are likely to continue reporting high levels of stress, burnout and suicide in situations of crisis (de Vibe et al., 2018). Mindfulness can have an important impact on healthcare professionals, improving their performance (Scheepers et al., 2020), wellbeing and mental health (Lomas et al., 2018), and promoting coping strategies that reduce stress (Burton et al., 2017). Qualitative findings related positive effects to specific mindfulness elements: self-awareness of stress and self-regulation of behaviors facilitated self-care of wellbeing (Verweij et al., 2016). When working in group, were doctors are used to share experience and promote peer support, mindfulness also appeared beneficial for wellbeing (Beckman et al., 2012; Verweij et al., 2016). In a previous study, GPs noticed that sharing experiences with peers helped them to deal with stressful events, by providing reassurance that they were not alone in their feelings (Beckman et al., 2012). This was particularly relevant to GPs working in solo practices, as usual in Italy and compared with ward nurses, working instead in group and with different frontline responsibilities compared to doctors with respect to patients. GPs reported that participation in a mindful-based intervention reduced their sense of professional isolation (Beckman et al., 2012). Our study evaluated the self-report measures of mindfulness compared with anxiety, stress and coping strategies of a representative sample of Italian GPs and nurses from L’Aquila city’s sanitary district. Questionnaires administration was undertaken during pandemic after the second wave of COVID-19 on spring 2021. L’Aquila city was already hit in the past by a sanitary emergency after the 2009 earthquake with 309 dead, 1,600 injured and approximately 80,000 displaced, with partial destruction of the city. The choice of this sample was also made to have a representation of the most significant moment in reorganized work responsibility for Italian GPs and hospital ward nurses with already a consolidated experience of extraordinary working conditions and traumatic stress due to a general emergency, and responses of this sensitive population were evaluated under pandemic.

## Materials and Methods

### Subjects

The research involved a total of 73 voluntary subjects aged between 23 and 69 years (see Supplementary Tables 1, 2 for a descriptive analysis of the sample). These subjects were taken from the sanitary district of ASL1 Avezzano-Sulmona-L’Aquila, in central Italy, as representative of a specific territorial context where the work organization of clinicians was rapidly reprogrammed for the emergency due to COVID-19 pandemic. GPs worked isolated in private ambulatories affiliated with the ASL1; nurses worked in specific and recently reorganized COVID-19 forefront hospital wards (Pulmonology, COVID-Medicine, Infectious diseases and Intensive care units). The number of voluntary subjects was 50% of total GPs and nurses working at that time in the district (88 GPs and 60 nurses); this allowed us to consider the recruited sample as representative not on the basis of specific control factors, but of that specific historical moment and territory in which the working reorganization due to COVID-19 occurred. Sample characteristics are provided in Table 1. The GPs sample consisted of 37 subjects aged between 27 and 69 years. The average seniority of GPs was 31.95 years of work activity (St Dev = 9.891), with a mean of 34.65 h per week (St Dev = 13.89). The nurse sample consisted of 36 subjects aged between 23 and 55 years. The average seniority of nurses was 6.39 years of work activity (St Dev = 8.23), with a mean of 36.17 h per week (St Dev = 0.85).

**TABLE 1:** Frequencies of responses (number of GPs and nurses subjects) and scoring to HPSCS questionnaire as divided for coping strategy.

	T scores	Solution of the problem		Request for social support		Emotional distress		Avoidance of the problem	
					
		GPs	Nurses	GPs	Nurses	GPs	Nurses	GPs	Nurses
Rarely/not at all	T < 35	5	1	10	1	1	4	2	0
Little	35 ≤ T < 45	9	4	10	5	9	13	11	9
On average	45 ≤ T < 55	11	18	14	11	11	13	15	10
Often	55 ≤ T < 65	11	9	3	14	14	3	8	5
Very frequently	T ≥ 65	1	4	0	5	2	3	1	12
*N* total		37	36	37	36	37	36	37	36

### Procedures

During the period from February to May 2021, GPs and nurses subjects completed a set of two self-report questionnaires under supervision. GPs were contacted telephonically to offer them to participate in this research, and then to arrange an appointment to personally administer the questionnaires in their private ambulatory. We obtained GPs contacts from the ASL1 database. In order to recruit nurses (personal contacts were not provided by ASL1), we contacted the primary and the head nurse of the respective hospital departments, which provided to contact the nurses. A psychologist from our group who already worked within those departments took care of personally administer and collecting the questionnaires from nurses at the ward. All the subjects were equally instructed to compile the questionnaires by using a standard Instructions form.

### Psychological Evaluation

Subjects were asked to compile the Cognitive and Affective Mindfulness Scale- Revised (CAMS-R), a questionnaire created by Feldman et al. (2006) to measure mindfulness as mindful attitude in stressful situations. The authors conceptualized mindfulness in a set of four attitude dimensions: attention, focus, acceptance and awareness. The CAMS-R questionnaire consists of 12 items, where the subject evaluates on a Likert scale ranging from 1 (“rarely/not at all”) to 4 (“almost always”) how much the statement is in accordance with their attitude. We administered the Italian adaptation of the CAMS-R (Veneziani and Voci, 2015).

Subjects were also asked to compile the Health Professions Stress and Coping Scale (HPSCS), a questionnaire for the evaluation of stress perceived in specific situations of healthcare professions and the use of coping strategies to reduce the stress (Spielberger et al., 1970). It proposes several potentially stressful work situations, and measures both the level of perceived stress as emotional exhaustion, depersonalization and personal fulfillment, and coping mechanisms used to cope with it. The HPSCS allows the assessment, for the individual or for an entire ward, of situations in which work efficiency is threatened and the risk of burn-out is looming, allowing to plan preventive and intervention focused strategies. In our case it was useful for detecting the experiences perceived as the most stressful and the strategies adopted to cope with them. It exists in two versions: one for doctors and one for nurses. The version for doctors consists of 23 items (situations) that refer to 5 areas, for a total of 115 statements. The areas are: personal attack and organizational contingencies, clinical emergency, confrontation with death, problematic relationships with patients, personal devaluation. The version for nurses consists of 19 items (situations) that refer to 5 areas, for a total of 95 statements. The areas are: clinical emergency, personal attack, organizational contingencies, personal devaluation, problematic relationships with patients and family members. Both versions can be used to measure perceived stress and coping strategies related to the totality of situations (Total Stress and General Coping Strategies), both to measure perceived stress and coping strategies in each of the five areas. Based on Lazarus and Folkman’s model (Lazarus and Folkman, 1984), HPSCS conceptualized coping by distinguishing 4 mechanisms of orientation: centered on the Solution of the problem (the most suitable solutions are sought with extensive use of technical knowledge), centered on the Request for social support (seeking for advice and help of other people), centered on Emotional distress (reacting on an emotional level and unable to properly manage and control emotions), or centered on Avoidance of the problem (attempting to avoid the problematic situation on a cognitive or behavioral level).

To understand the effects of mindful attitude on reaction to anxiety and consequent coping strategies, we administered the State-Trait Anxiety Inventory in the form X2 (STAI-X2) assessing the trait emotion, or relatively stable individual characteristics as regarding predisposition to anxiety (Spielberger et al., 1970, 1980). Particularly, trait anxiety indicates that tendency to perceive situations which are even objectively not dangerous as threatening, responding to them with an anxiety that is disproportionate to the severity of the danger. The test consists of 20 questions for each of which there are 4 possible answers, with the relative scores on a Likert scale ranging from 4 (“almost always”) to 1 point (“almost never”). For some responses, the scores must be reversed as the questions refer to the absence of anxiety.

### Data Analysis

The CAMS-R and STAI-X2 questionnaires were entered directly into Excel tables for cleaning and scoring, instead the HPSCS data were entered in insertion tables on the Giunti editor’s website, as requested by the publisher, necessary for restitution of standard protocol.

According to the CAMS-R questionnaire manual (Feldman et al., 2006; Veneziani and Voci, 2015), there are some negative items that require to be reversed before proceeding with the statistical analysis: the scoring of items 2, 6, and 7 have therefore been converted (1 = 4; 2 = 3; 3 = 2; 4 = 1). Descriptive statistics were carried out calculating the sum for each subject, and the mean. The reliability analysis of the questionnaire was carried out by means of SPSS software and applying Cronbach’s alpha index. This gave a result of ≥ 0.6 making the questionnaire reliable. As authors of the CAMS-R, Feldman et al. (2006) provided 4 factors resulting from the questionnaire factor analysis: Attention (items 1, 6, 12), Focus (items 2, 7, 11), Awareness (items 5, 8, 9), and Acceptance (items 3, 4, 10). By means of SPSS, clusters were created to isolate these factors through the calculation of variables.

About the HPSCS questionnaires, after entering the data obtained from the reports in SPSS, the properties of these questionnaires allowed us to move quite freely in the organization of data analysis. First of all, a reliability analysis (Cronbach’s alpha) was calculated with respect to the areas proposed by the questionnaire. Unfortunately it turned out to be ≤ 0.6 and consequently not reliable. Reliability (Cronbach’s alpha) was then assessed taking into account the different coping strategies. In fact, each situation presented 4 alternatives, equivalent to the 4 coping strategies originally identified by Lazarus and Folkman (1984), on the basis of the theoretical criteria of the questionnaire: Solution of the problem, Request for social support, Emotional distress, and Avoidance of the problem. As demonstrated by internal consistency and item total correlation coefficients relating to the coping strategies present in the HPSCS editor’s manual (Ripamonti et al., 2008) Cronbach’s alpha resulted ≥ 0.6.

The STAI-X2 trait anxiety questionnaire presented negative questions to be reversed as above mentioned for the CAMS-R. The analysis was carried out according to the normative tables of the editor’s manual (Sanavio et al., 1998), by calculating sums and averages of Z scores, even through the calculation of the ds *Z* = (raw-average score)/ds. The reliability analysis of the questionnaire was carried out with SPSS, applying Cronbach’s alpha, resulting in ≥ 0.6 making the questionnaire reliable.

Correlations were assessed (bivariate Pearson’s correlation) by means of SPSS software and taking into account all three questionnaire tests. For HPSCS, reliable results were directly taken as calculated for the 4 coping strategies). Subsequently, specific correlations were calculated between the factors of CAMS-R and the coping strategies of the HPSCS.

## Results

### Instruments Reliability Criteria

HPSCS reliability (Cronbach’s alpha) was assessed for all the four coping strategies: Solution of the problem (0.814), Request for social support (0.898), Emotional distress (0.855), and Avoidance of the problem (0.778). CAMS-R reliability (Cronbach’s alpha) was also assessed upon the sum of all coping strategies (0.724). STAI-X2 reliability (Cronbach’s alpha) was also calculated in a test-retest correlation with a range from 0.73 to 0.86, while the internal consistency index varying in the range between 0.86 and 0.92 (Cronbach’s alpha).

### Comparative Analysis

Reference values of anxiety as measured by means of STAI-X2 scale were taken from CBA 2.0 battery test (Sanavio et al., 1998), depending on the sex and age of the subjects. As obtained from this analysis, the 75.7% of GPs had normal anxiety as average levels, 13.5% had low and 10.8% had high anxiety levels, indicating a not critical impact of the stress on trait anxiety at this point of the pandemic. Compared with GPs, a relevant quote of nurses had lower levels of anxiety since 50% of nurses had low levels of anxiety, and 47.22% had normal but only 2.78% had high levels of anxiety. Taken together, although not demonstrating an acute phase for GPs, these results show that a greater number of them incorporated anxious traits and attitudes related to stress, probably due to the different role and organizational context of isolation in which they had to work in recent times of pandemic.

Table 1 shows results of GPs and nurses at the HPSCS questionnaire. Solution of the problem, Request for social support, Emotional distress, and Avoidance of the problem were the four coping strategies considered from the test. This analysis points out to the tendency for using the “Emotional distress” strategy with higher frequency in GPs, but difference was not significant as this strategy aligned with the norm (mean T scores = 50, StDev = 10). At difference, nurses had the tendency for using the “Avoidance of the problem” and the “Request for social support” strategies with significantly higher frequencies, and “Emotional distress” with significantly lower frequency. Compared with GPs, these results suggest a different pattern of coping in clinical practice between GPs and nurses.

HPSCS test resulted also in a quantification of the level of stress perceived. This analysis suggests that GPs were moderately stressed (32,43% of subjects on average stress levels), with a similar number of subjects with low (16,22%), very low (18,92%) or high (18,92%), and very high (13,51%) stress. Compared with GPs, stress levels measured from HPSCS test in nurses were lower, with only 5.56% of nurses with high and 2.78% with very high levels, and most of nurses on average (36,11%), low (30,55%) or very low (25%) levels of stress. No substantial difference in responses between subject sex or age allowed to make further assumptions (data not shown), suggesting for a more detailed comparative analysis to be performed.

Table 2 shows results of an independent sample Levene’s test and Student’s *t*-test for comparison between variances and means of CAMS-R test scores of GPs in the different four factors as coping strategies of mindfulness: Attention, Focus, Awareness and Acceptance. Mean comparisons were not significant as showing no difference between the four different CAMS-R coping strategies and the mean of all CAMS-R scores was 3.08 with a StDev of 0.39. This legitimated us to compare each different CAMS-R factor to HPSCS test’s coping strategies and STAI-X2 anxiety as subscales.

**TABLE 2:** Analysis of variance between the means of CAMS-R test scores of GPs in the single test (CAMS) and in the four factors (attention, focus, awareness, acceptance) by means of Levene’s test and student’s *t*-test for independent samples (upper row assumed equal variances, lower row not assumed equal variances).

						*t*-test for the equality of means					
						
				Levene’s test for the equality of variances						Confidence interval of difference 95%	
									
	Mean	StDev	Mean St.Error	*F*	Sign.	*t*	df	Sign.	Mean dif.	Inferior	Superior
Acceptance	2.963	0.562	0.093	0.662	0.421	−0.25	35	0.804	0.04969	−0.4535	0.3542
						−0.259	30.918	0.797	−0.04969	−0.4402	0.3409
Awareness	2.888	0.596	0.099	1.471	0.233	0.244	35	0.809	0.05176	−0.379	0.4825
						0.223	20.489	0.826	0.05176	−0.4323	0.5358
Focus	3.129	0.565	0.094	1.489	0.231	1.205	35	0.236	0.19462	−0.1331	0.5224
						1.137	22.758	0.268	0.19462	−0.1598	0.549
Attention	3.49	0.454	0.075	0.411	0.526	−0.162	35	0.872	−0.02899	−0.3923	0.3343
						−0.171	32.301	0.865	−0.02899	−0.3737	0.3157

Table 3 shows results of an independent sample Levene’s test and Student’s *t*-test for comparison between variances and means of CAMS-R test scores of nurses in the different four factors as coping strategies of mindfulness: Attention, Focus, Awareness and Acceptance. Mean comparisons were not significant as showing no difference between the four different CAMS-R coping strategies and the mean of all CAMS-R scores was 3.12 with a StDev of 0.42. This legitimated us to compare each different CAMS-R factor to HPSCS test’s coping strategies and STAI-X2 anxiety as subscales.

**TABLE 3:** Analysis of variance between the means of CAMS-R test scores of nurses in the single test (CAMS) and in the four factors (attention, focus, awareness, acceptance) by means of Levene’s test and student’s *t*-test for independent samples (upper row assumed equal variances, lower row not assumed equal variances).

						*t*-test for the equality of means					
						
				Levene’s test for the equality of variances						Confidence interval of difference 95%	
									
	Mean	StDev	Mean St. Error	*F*	Sign.	*t*	df	Sign.	Mean dif.	Inferior	Superior
Acceptance	2.963	0.562	0.093	0.186	0.669	-0.075	34	0.941	0.18181	-0.383	0.3559
						-0.085	10.861	0.934	0.15996	-0.3661	0.339
Awareness	2.888	0.596	0.099	0.803	0.376	0.212	34	0.834	0.19392	-0.353	0.4351
						0.187	8.054	0.856	0.21977	-0.4651	0.5472
Focus	3.129	0.565	0.094	0.044	0.835	1.325	34	0.194	0.2354	-0.1664	0.7903
						1.27	8.71	0.237	0.24574	-0.2467	0.8707
Attention	3.49	0.454	0.075	1.28	0.266	-0.301	34	0.765	0.23988	-0.5597	0.4152
						-0.402	14.83	0.693	0.17951	-0.4552	0.3107

### Correlational Analysis

Analysis of correlation between trait anxiety and mindful attitude in GPs, represented in Table 4, demonstrated a negative correlation between anxiety and mindfulness as measured, respectively, by means of STAI-X2 and CAMS-R tests (*p*, 0.01 at a bilateral level). This indicates that predisposition of mindful positive strategies has lowered anxiety in GPs. Interestingly, anxiety was positively correlated with coping strategies as measured by means of the HPSCS test, in particular with Request for social support (*p*, 0.05 at a bilateral level), suggesting that GPs may have suffered stress for isolation when activating for requesting more social support. Emotional distress was not correlated to anxiety, but positively correlated to Request for social support and to Avoidance of the problem (*p*, 0.01 at a bilateral level), indicating a complementary activation of negative coping strategies to stress and negative emotions. Request for social support also positively correlated with Avoidance of the problem (*p*, 0.05 at a bilateral level). Conversely, mindful attitude positively correlated with Solution of the problem (*p*, 0.05 at a bilateral level), indicating that those mindful GPs activated a positive coping strategy to address the problem for its solution.

**TABLE 4:** Correlations between trait anxiety and mindful attitude in GPs, calculated as bivariate Pearson’s correlation of the sum of, respectively, of STAY-X2 and CAMS-R scores, and with the sum of each HPSCS coping strategy scores.

				HPSCS coping strategies			
				
		STAY-X2	CAMS-R	Solution of the problem	Request for social support	Emotional distress	Avoidance of the problem
	STAY-X2	1	−0.514**	−0.063	0.380*	0.282	0.242
	CAMS-R	−0.514**	1	0.366*	−0.032	0.086	−0.044
HPSCS coping strategies	Solution of the problem	−0.063	0.366*	1	0.233	0.079	−0.214
	Request for social support	0.380*	−0.032	0.233	1	0.640**	0.400*
	Emotional distress	0.282	0.086	0.079	0.640**	1	0.468**
	Avoidance the problem	0.242	−0.044	−0.214	0.400*	0.468**	1

**Correlation is significant at the 0.01 level (2-tailed). *Correlation is significant at the 0.05 level (2-tailed).

By confronting the GPs’ mindful coping strategies measured by means of the CAMS-R test factors with positive or negative coping strategies as measured by means of the HPSCS test (Table 5), we found a negative correlation between Focus and not conclusive coping strategies as Request for social support and Avoidance of the problem (p, 0.05 at a bilateral level), indicating that personal mindful predisposition of GPs activated Focus strategy to cope to stress instead of requesting for social support and trying to avoid the problem. At same time, we found a positive correlation between Attention and positive coping strategy as Solution of the problem, confirming that mindful GPs activated resolutive coping.

**TABLE 5:** Correlations of mindful attitude (CAMS-R) and mindful coping strategies (awareness, acceptance, focus, attention) with HPSCS coping strategies in GPs, calculated as bivariate Pearson’s correlation of the sum of, respectively, of CAMS-R and each factors’ scores with the sum of each HPSCS coping strategy scores.

		HPSCS coping strategies			
		
		Solution of the problem	Request for social support	Emotional distress	Avoidance of the problem
CAMS-R factors	Awareness	0.304	0.228	0.285	0.147
	Acceptance	0.234	0.072	0.108	0.18
	Focus	0.077	−0.409*	−0.298	−0.361*
	Attention	0.421**	−0.071	0.078	−0.178

**Correlation is significant at the 0.01 level (2-tailed). *Correlation is significant at the 0.05 level (2-tailed).

Tables 6, 7 show results of the same correlation analyses on nurses. Similarly to GPs (Table 4), correlation between trait anxiety and mindful attitude in nurses represented in Table 1 demonstrated a negative correlation between anxiety and mindfulness as measured, respectively, by means of STAI-X2 and CAMS-R tests (*p*, 0.01 at a bilateral level). This indicates that predisposition of mindful positive strategies has lowered anxiety also in nurses. Anxiety was strongly positively correlated with Request for social support (*p*, 0.01 at a bilateral level), but since levels of anxiety and stress were lower in nurses than in GPs as showed before, we suggest that nurses activated for requesting social support from doctors or other ward nurses as normally they do to cope with problematic situations. On the other hand, emotional distress was not correlated to anxiety, but positively correlated to Request for social support and to Avoidance of the problem (*p*, 0.01 and *p*, 0.05, respectively, at a bilateral level), indicating a complementary activation of negative coping strategies to stress and negative emotions similarly to GPs (Table 4). Interestingly, mindful attitude in nurses activated coping strategy different from GPs, as positively correlating with Avoidance of the problem (*p*, 0.01 at a bilateral level), indicating that those mindful nurses activated a coping strategy to not personally address the problem.

**TABLE 6:** Correlations between trait anxiety and mindful attitude in nurses, calculated as bivariate Pearson’s correlation of the sum of, respectively, of STAY-X2 and CAMS-R scores, and with the sum of each HPSCS coping strategy scores.

				HPSCS coping strategies			
				
		STAY-X2	CAMS-R	Solution of the problem	Request for social support	Emotional distress	Avoidance of the problem
	STAY-X2	1	−0.641**	−0.138	0.556**	0.321	0.026
	CAMS-R	−0.641**	1	0.216	−0.094	0.081	0.442**
HPSCS coping strategies	Solution of the problem	−0.138	0.216	1	0.072	−0.249	−0.126
	Request for social support	0.556**	−0.094	0.072	1	0.518**	0.305
	Emotional distress	0.321	0.081	−0.249	0.518**	1	0.351*
	Avoidance the problem	0.026	0.442**	−0.126	0.305	0.351*	1

**Correlation is significant at the 0.01 level (2-tailed). *Correlation is significant at the 0.05 level (2-tailed).

**TABLE 7:** Correlations of mindful attitude (CAMS-R) and mindful coping strategies (Awareness, Acceptance, Focus, Attention) with HPSCS coping strategies in nurses, calculated as bivariate Pearson’s correlation of the sum of, respectively, of CAMS-R and each factors’ scores with the sum of each HPSCS coping strategy scores.

		HPSCS coping strategies			
		
		Solution of the problem	Request for social support	Emotional distress	Avoidance of the problem
CAMS-R factors	Awareness	0.326	−0.032	0.164	0.510**
	Acceptance	0.088	0.092	0.177	0.400*
	Focus	0.071	−0.326	−0.091	0.132
	Attention	0.183	−0.018	−0.016	0.326

**Correlation is significant at the 0.01 level (2-tailed). *Correlation is significant at the 0.05 level (2-tailed).

Moreover, Avoidance of the problem in nurses was positively correlated with mindful coping strategies of Awareness (*p*, 0.01 at a bilateral level) and Acceptance (*p*, 0.05 at a bilateral level), confirming that nurses activated a different, not resolutive but even mindful, pattern of coping (Table 7).

## Discussion

In this study we wanted to evaluate anxiety and stress as perceived by GPs working alone in ambulatory practice room and by hospital ward nurses during a chronic phase of COVID-19 pandemic sanitary emergency (February–May 2021). Subjects were taken from the suburban area of a small Italian town, L’Aquila, where citizens and health professionals were already sensitive to sanitary emergency with great psychosocial effects as for the recent earthquake on 2009. Subjects could have eventual post-traumatic stress long-term effects, but even formed experience of coping in a situation of extraordinary work reorganization and with measures to contain distress. The choice of this sample was made in order to highlight the possible effects of traumatic stress on coping strategies aimed at solving problems due to the professional role, not only for the risk of being at the first touchpoint with the patients, but also to the actual reorganization that these have undergone as imposed by pandemic from national health system. Since GPs work individually, and nurses work in teams, the configuration of the sample groups could almost partially account for the differences found in results, beyond mindfulness practices and anxiety levels. Recent studies showed that healthcare workers directly involved with COVID-19 patients and from areas with higher rates of contagion reported higher levels in perceived stress, anxiety, depression, burnout, and secondary trauma levels than their colleagues working with non-COVID-19 patients (Trumello et al., 2020; Buselli et al., 2020; Dobson et al., 2021). In this context, organizational support systems can be an invaluable buffer against burnout and increase employee perceptions of their own health, for example the presence of coworker support aided in reducing perceptions of disengagement in team nurses (Finuf et al., 2021). When team members felt they had support from both their coworkers and supervisor, disengagement was significantly reduced compared to coworker support alone; moreover the lower emotional exhaustion translated to increased perceptions of both physical and psychological health. Burnout is negatively correlated with perceived organizational support and coworker social support in nurses (Lowe et al., 2020). Therefore we can imagine that GPs, working alone and without working support conditions, have been more severely exposed to the consequences of pandemic.

Our analyses showed that GPs were generally not in burden for stress and anxiety on that phase of emergency, but comparisons to nurses showed a higher number of GPs with high levels of anxiety and stress, and higher tendency to use emotional distress as coping strategy. These tendencies could be confirmed as significant if using a larger study sample. Therefore, coping strategies provide information to determine changes to intermediate or low levels of stress and anxiety. Comparisons with mindfulness coping strategies allowed to understand the effects of each coping strategy on reducing stress and anxiety at this chronic phase. We found that anxiety induced by a threatening and traumatic adaptive situation such as pandemic was significantly reduced in both GPs and nurses with mindful attitude, with mindfulness as protective for anxiety. As anxiety increased both in GPs and nurses, this was coped by increasing the demand for social support. This coping strategy correlated with emotional distress and when enhanced, emotional distress corresponded to the avoidance of the problem. Since social support positively correlated with avoidance of the problem, we suggest that GPs may have engaged in seeking social support, for example with colleagues from hospital departments, in order to avoid the problem of the patients instead of addressing it. Both GPs and nurses coping strategies suggest that when present, a mindful attitude triggered analogous responses to anxiety and stress, but addressed to different targets into the organizational care context and on the basis of the role and skills of the investigated health personnel. We can suggest that nurses were accustomed to entrusting the problem to a doctor or to delegating the competent staff of the ward. This difference in mindful attitude of nurses can be traced back in the current primary care situation to the different role and working environment compared to the GP.

To better understand how mindful attitude determined the coping responses of GPs as compared with nurses, we must refer to the extraordinary changes that pandemic induced in the different organizational contexts of GPs compared to hospital ward nurses. A recent national survey studied between May and June 2021 the changes in professional role and relations of GPs toward citizens as induced by pandemic (Quotidianosanità, 2021). Some statistics from this survey are relevant to understand the psychosocial background of our sample: 55.8% of citizen sample reported that “personal GPs is special and would not want to change it,” more than 50% having a relationship with the GP longer than 10 years, 22.6% longer than 20 years, with generally high satisfaction for availability (78.9%) and contactability (75.1%) of personal GP, these confirming the long-time personal relationship that patients have with own GP. In case of need for a medical consultation, 77.4% of citizens contact primarily the personal GP, confirming the priority of GPs at the forefront of pandemic. On the other side, 79% of interviewed GPs reported that trust relationship with patients was changed compared to the past as mostly due to technology and emergent needs for reorganization of the national health system, 55.8% reporting this translating into a negative change for the GP’s profession, and 53.4% unsatisfied of territorial primary healthcare organization during the pandemic. Particularly, 83.7% felt not supported by local health institutions during the pandemic months, and 94.8% felt like a “hero” in this period.

In GPs, negative coping strategies were prevented by mindful attitude which lead to the solution of the problem as positive coping strategy, and this associated to attention and focus as mindful coping mechanisms. Differently, awareness and acceptance as mindful attitudes in nurses correlated with the avoidance of the problem. This major difference result suggests that nurses, differently from GPs, precisely because they accepted and consciously addressed the problem to others, avoiding solving it themselves as beyond their role and organizational responsibility. On the contrary the doctor had responsibility to provide by himself diagnostic and therapeutic responses for the patient, and this responsibility was taken over in case of the GP. In fact, GPs along with the demand for social support, focused on the problem trying to solve it by themselves, when predisposed by their mindful attitude. In this case we can assume that they put attention to the problem until its solution and without avoiding it.

Taken together, these results suggest that GPs took charge of the problem positively and tried to solve it by referring the patient to the competent structures where they were not able to treat them in their own. It is very important to underline that mindful attitude in GPs is the only one that leads to the solution of the problem, by taking place specifically through greater attention to the patient’s request as a unique mindful coping strategy. On the other side, providing an important ward organization and the possibility to cope together with doctors and colleagues outlines a different context allowing for implementation of mindful coping strategies in nurses.

Doctors experience mindfulness as helping them to become more aware of stress, proactively set priorities and limits, and develop a healthier relationship to work in a stressful practice environment (Verweij et al., 2018). On the other side, a good group organization is the preeminent work environment for developing mindful attitude in nurses, since they experience considerable job stress as a major factor in the high rates of burnout (Shimizu et al., 2003), and work related stress leads to a much higher turnover, especially during the first years post-graduate, lowering retention rates in general (Zangaro and Soeken, 2007; Botha et al., 2015). Comparing with GPs, mindfulness is most effective when embedded in an organizational approach that promotes a culture of wellness to doctors in various educational phases (from post-graduate to continuing medical education), when also addressing system-related demands and workloads (Panagioti et al., 2017; Shanafelt and Noseworthy, 2017; Panagopoulou and Montgomery, 2019). When hospital-based, doctors reported positive effects of group-based mindfulness interventions on their wellbeing with respect to anxiety and stress, or burnout and empowerment at work (Sood et al., 2011, 2014; West et al., 2014; Amutio et al., 2015; Pflugeisen et al., 2016). Working in solo, GPs similarly reported positive effects on psychological or occupational wellbeing (Krasner et al., 2009; Verweij et al., 2016; Hamilton-West et al., 2018). Qualitative findings related positive effects to specific mindfulness elements in GPs: self-awareness of stress and self-regulation of behaviors facilitated self-care of wellbeing (Verweij et al., 2016). However, a group-based setting promoting shared experience and peer support also appeared beneficial for wellbeing, and reducing GPs’ sense of isolation from other physicians, nurses and health system in general (Beckman et al., 2012; Verweij et al., 2016). The GP’s sense of responsibility therefore appears to be a determining factor in responses to anxiety and stress on emergency situations as pandemic, and in addressing coping strategies inside an organizational context where he had to decide alone for diagnosis and fate of his patients.

We suggest that self-awareness as the mindful attitude of attention to the problem could have adaptively addressed the sense of responsibility as the emotional correlate of anxiety in GPs. We know that adaptive or physiological anxiety prepares for potential danger and can contribute to coping with difficult situations (Perilli et al., 2020). It is defined pathological when occurring in inappropriate contexts with an excessive intensity on the continuum from moderate to extreme. Trait anxiety as measured in this study is the component inherent in the individual predisposition to respond to situation with more or less anxiety. Mindful attitude is a disposition that individuals can present to a greater or lesser extent, regardless of mindfulness practice. As the mind can be focused, disturbed, dreaming, slowed down, etc., it can also be mindful (Bruce et al., 2010). Even the practice of mindfulness, by training people to cultivate awareness for increasing periods of time and with constancy, can allow them to achieve a more mindful attitude and focus the problems toward their resolution, by reducing the personal predisposition to stress and burnout, and modulating responses with positive emotions (Perilli et al., 2020). A mindful attitude may be able to address, at least in part, GPs’ current challenges in finding meaning in their work and connecting with patients in isolated and stressful practice environments as induced by pandemic reorganization (Shanafelt et al., 2012; Zhu et al., 2021). After mindfulness training, doctors reported enhanced connection with patients as a better ability to listen deeply, be attentive to patients’ concerns and effectively respond to their request (Krasner et al., 2009; Beckman et al., 2012; Lases et al., 2016; Bentley et al., 2018; Verweij et al., 2018). Patients positively recognized this attitude reporting that mindful clinicians (both doctors and nurses) communicated in a more patient-centered way, and engaged to a greater degree in psychosocial relationship (Beach et al., 2013).

Mindfulness-based interventions may therefore be proposed to enhance doctors’ and nurses’ empathic care, as well as for prevention of stress and burnout under reorganization of primary care as due to a sanitary emergency. In our study we demonstrated that GPs and nurses primarily activated the Request for social support as coping strategy. At this point in the pandemic, we can ask ourselves what social support tools were put in place by national health and social institutions to assess their impact on stress management in primary care clinicians. Psychological support interventions and assistance desks were activated and made available to clinicians as well as patients, but the conditions of isolation and the new rules of social distancing not always allowed these interventions to be effective in managing burning anxiety and stress. Psychologists themselves as doctors, have been subject to a drastic reorganization of their professional practice, many of them temporarily withdrawing for not being more able to receive patients give their support in effective way. Mindfulness-based interventions could be combined with organizational changes to promote a supportive work environment that reinforces and does not undermine the emergency measures. Mindfulness is to develop individual skills to manage stress, such as knowing how to listen and pay attention to the problem, to express emotions, to accept pain and knowing how to communicate with the patient and with the colleagues, to move in difficult contexts and solve extraordinary problems posed by the emergency. A mindful attitude can therefore prevent the chronicization of anxiety and the dysfunctional reactive behaviors in the GP, which operates alone in the context of the pandemic, as well as in the nurse subject to increased stress reaction for the victims and for anguish and aggression of hospitalized patients, by enhancing comfort and emotional support. The expression of emotions in an emergency context can be facilitated through the acquisition of the ability to understand and deal with the strong emotional reactions connected to the traumatic event.

Our findings together with other promising on the long-term effects of mindfulness-based interventions (de Vibe et al., 2018) quote for intensified research on the effectiveness of incorporating mindfulness as a parameter into medical and nurse schools’ curricula in order to equip graduates with skills that can be sustained throughout their clinical careers and that could be crucial for dealing with emergencies and to forefront pandemics.

## Data Availability Statement

The raw data supporting the conclusions of this article will be made available by the authors, without undue reservation.

## Ethics Statement

The studies involving human participants were reviewed and approved by Internal Review Board (IRB) of the University of L’Aquila, Italy (Prot. No. 107751/2020). The patients/participants provided their written informed consent to participate in this study.

## Author Contributions

EP: conceptualization and project administration. MP, DB, and SM: methodology. MP and SM: software. EP, MP, and DB: validation. MP, DB, SM, and SC: formal analysis. MP, DD, and CC: resources. EP and SM: data curation. EP and SC: writing—original draft preparation. EP, MP, DB, FR, and SC: writing—review and editing. EP and MP: visualization. EP, DD, and SC: supervision. All authors contributed to manuscript revision, read, and approved the submitted version.

## Conflict of Interest

The authors declare that the research was conducted in the absence of any commercial or financial relationships that could be construed as a potential conflict of interest.

## Publisher’s Note

All claims expressed in this article are solely those of the authors and do not necessarily represent those of their affiliated organizations, or those of the publisher, the editors and the reviewers. Any product that may be evaluated in this article, or claim that may be made by its manufacturer, is not guaranteed or endorsed by the publisher.
