# Supplementary material for: Reduced Anxiety Associated to Adaptive and Mindful Coping Strategies in General Practitioners Compared With Hospital Nurses in Response to COVID-19 Pandemic Primary Care Reorganization
Source: Front Psychol. 2022 Jun 9;13:891470. doi: 10.3389/fpsyg.2022.891470 (PMC9218856; doi:10.3389/fpsyg.2022.891470)
Supplement: Supplementary file 1 [file Table_1.docx]

**Supplementary Material**

Table 1. Descriptive analysis of the sample divided into total GPs and nurses.

|  | | Age | | Professional seniority | | Present work place seniority | | Weekly work hours | |
| --- | --- | --- | --- | --- | --- | --- | --- | --- | --- |
|  |  | GPs | Nurses | GPs | Nurses | GPs | Nurses | GPs | Nurses |
| N | | 37 | 36 | 37 | 36 | 37 | 36 | 37 | 36 |
| Mean | | 61,946 | 31,97 | 31,946 | 6,39 | 26,514 | 2,14 | 34,649 | 36,17 |
| Median | | 64,000 | 29,00 | 33,000 | 2,50 | 30,000 | 1,00 | 30,000 | 36,00 |
| Mode | | 68,0 | 24 | 30,0^a^ | 1 | 30,0^a^ | 1 | 30,0 | 36 |
| std.Dev. | | 9,0583 | 8,927 | 9,8909 | 8,233 | 11,4203 | 2,474 | 13,8949 | ,845 |
| Variance | | 82,053 | 79,685 | 97,830 | 67,787 | 130,423 | 6,123 | 193,068 | ,714 |
| Minimum | | 27,0 | 23 | 1,0 | 1 | 1,0 | 1 | 15,0 | 35 |
| Maximum | | 69,0 | 55 | 45,0 | 34 | 45,0 | 12 | 64,0 | 40 |
| a. Different modalities. Smaller value is showed | | | | | | | | |  |
