# Supplementary material for: Reduced Anxiety Associated to Adaptive and Mindful Coping Strategies in General Practitioners Compared With Hospital Nurses in Response to COVID-19 Pandemic Primary Care Reorganization
Source: Front Psychol. 2022 Jun 9;13:891470. doi: 10.3389/fpsyg.2022.891470 (PMC9218856; doi:10.3389/fpsyg.2022.891470)
Supplement: Supplementary file 2 [file Table_2.docx]

**Supplementary Material**

Table 2. Sex distribution in the sample divided into GPs and nurses.

|  | Frequency | | Percentual | |
| --- | --- | --- | --- | --- |
|  | GPs | Nurses | GPs | Nurses |
| Male | 23 | 7 | 62,2 | 19,4 |
| Female | 14 | 29 | 37,8 | 80,6 |
| Total | 37 | 36 | 100,0 | 100,0 |
